# *Moringa Oleifera* leaf extract induced pulmonary embolism-a case report

**DOI:** 10.1186/s12245-022-00419-3

**Published:** 2022-04-12

**Authors:** Ebehiwele Ebhohon, Dwayvania Miller

**Affiliations:** Department of Internal Medicine, Lincoln Medical Center, 234 E 149 St, Bronx, NY 10451 USA

**Keywords:** *Moringa oleifera*, Venous thromboembolism, Pulmonary embolism, Deep venous thrombosis

## Abstract

**Background:**

*M. oleifera* leaf extract supplement is famous for its anti-inflammatory, antioxidant, antimicrobial, antifertility, anticancer, antihepatotoxic, and antiulcer properties. However, limited data exist on the coagulation effect of *M. oleifera* leaf extract in human plasma, which maybe a predisposing factor to venous thromboembolism (DVT and PE); a disorder that is well known to be induced by risk factors such as surgery, trauma, cancer, or prolonged immobility.

**Case presentation:**

We report a case of a 63-year-old Hispanic female with past medical history of obesity and type 2 diabetes mellitus who presented to the emergency room with a three-day history of worsening shortness of breath and chest pain. Computerized tomography-pulmonary angiogram (CT-PA) revealed bilateral pulmonary embolism (PE) and right ventricle strain. Based on CT imaging findings, the absence of a major transient risk factor for venous thromboembolism (VTE), no history suggestive of an underlying hypercoagulable disorder, and a medication history that was significant for a recent 5-month use of *M. oleifera* leaf extract that has been reported to induce clot formation, she was diagnosed as a rare case of sub-massive pulmonary embolism provoked by *M. oleifera* leaf extract supplement. She received initial anticoagulation (AC) during her hospitalization and was discharged on maintenance AC for 3 months.

**Discussion and conclusion:**

We report the first case of PE likely triggered by using *Moringa oleifera* leaf extract herbal supplement. Cohort studies on the coagulation effect of *Moringa oleifera* leaf extract in humans are necessary to determine the relationship between *Moringa Oleifera* leaf extract and VTEs.

## Background

Venous thromboembolism (VTE) is a term that includes a spectrum of conditions from asymptomatic deep venous thrombosis (DVT) to fatal pulmonary embolism (PE). PE accounts for 100,000 to 200,000 deaths in the USA annually [1, 2]. The clinical presentation of Pulmonary Embolism (PE) varies, making the diagnosis habitually challenging and requiring a high index of suspicion. VTE may be caused by an acquired risk factor (provoked event) or can occur in the absence of any provoking risk factor (unprovoked event). Common risk factors for VTE include prior thromboembolism, age > 60 years, surgery, trauma, cancer, reduced immobility, etc. Therefore, recognizing the etiology or risk factors for VTE is necessary to determine the duration of anticoagulation therapy; typically, 3 or 6 months if provoked; and lifelong anticoagulation if unprovoked.

*Moringa oleifera*, a large tree that is widely cultivated in India, Asia, Africa, and America is commonly believed to be rich in proteins, vitamin A, minerals, and isothiocyanates that exhibit anti-inflammatory, antioxidant, anticancer, hepatoprotective, neuroprotective, anti-hyperglycemic, and blood lipid-reducing properties [3]. As a result of its properties, it is generally believed to manage several chronic conditions such as hypercholesterolemia, hypertension, diabetes, cancer, and inflammation [4], Additionally, it has been widely used in purifying water and traditionally to accelerate wound healing, resulting in its growing significance [4–9]. However, there is limited scientific data about *M. oleifera* coagulation properties that can activate the human blood coagulation cascade [9, 10]. Therefore, our case report describes a rare incidence of pulmonary embolism likely precipitated by the prolonged use of *M. oleifera* leaf extract in a patient with low risk for venous thromboembolic events.

## Case presentation

**Fig. 1:**
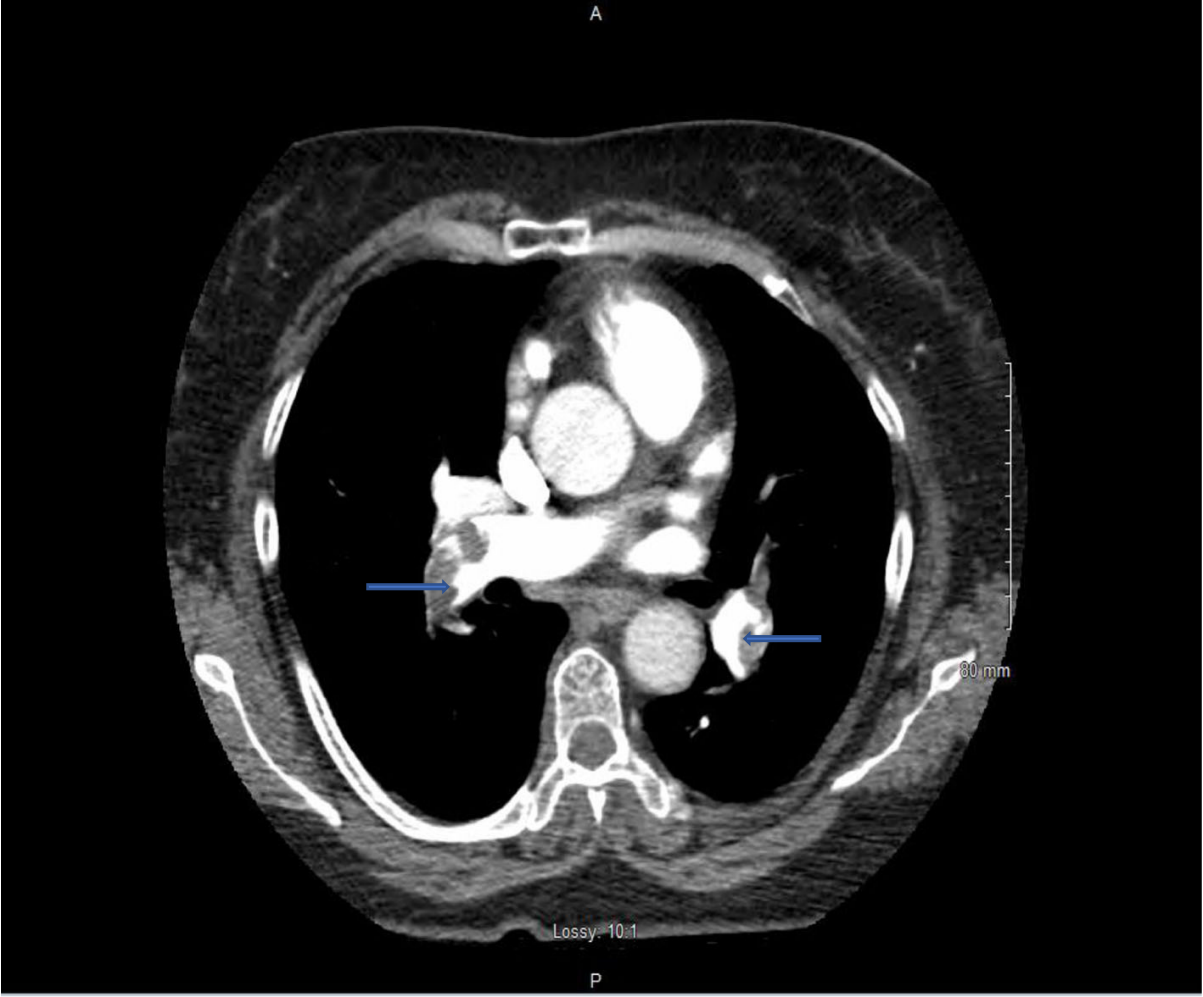
CT scan demonstrates regions of hyper-attenuation within the right main and left pulmonary arteries (arrowheads)

**Fig. 2:**
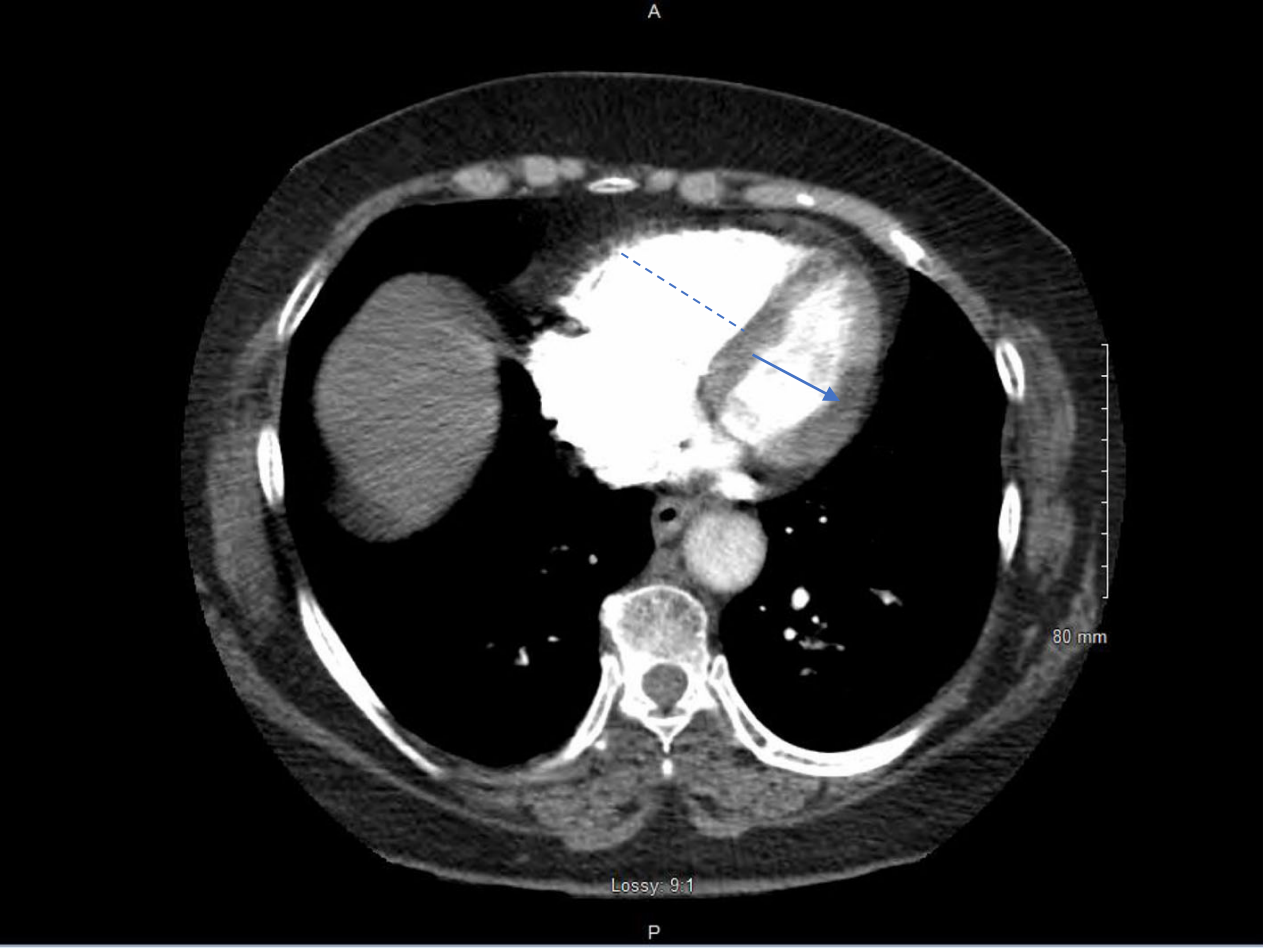
CT scan shows the short axis of the right ventricle (dashed line) is wider than the left ventricle (solid line), a condition called RV strain and is caused by acute pulmonary embolism

A 63-year-old Hispanic female (height 1.5 m, weight 76.1 kg, BMI 33.8 kg/m^2^) and lifetime non-smoker who was admitted to the ER with 3 days of shortness of breath and chest pain that worsened on ambulation. Her medical history included obesity and type 2 diabetes for which she was on metformin. She also reported a 5-month history of *M. oleifera* leaf extract supplement use. Her vitals were normal except for tachypnea and low oxygen saturation (93%) on room air. She spoke in complete sentences and there was no use of accessory muscles. Chest was clear bilaterally with no abnormal heart sounds. She had a slightly larger right lower extremity calf diameter when compared to the left lower extremity. Arterial blood gas showed increased A-a gradient, hypocapnia, and hypoxemia. CXR and EKG were normal. PoCUS revealed right ventricle (RV) dilation and moderately reduced cardiac contractility. An emergent CTPA showed bilateral PE with RV strain (Figs. 1 and 2). She was started on initial anticoagulation (AC) and admitted to the ICU for hemodynamic monitoring. Venous Duplex US lower extremity bilateral was negative for DVT. We did not proceed with catheter-directed thrombolysis since she was hemodynamically stable, improved with supplemental oxygen, and had no contraindication to systemic fibrinolysis. Upon further evaluation, her history was negative for any major transient risk factor for VTE. Similarly, her up-to-date mammogram, colonoscopy, pap smear, and intravaginal ultrasound were negative for malignancy. Since she had a negative personal and family history of VTE and no obvious risk factors for the event, we determined that the sub-massive PE was likely induced by *Moringa Oleifera* leaf extract that was documented by two studies as having procoagulant effect in human plasma. So, she was advised to discontinue use of the supplement. After 72 hours, she was discharged on maintenance AC for 3 months with primary care follow-up given. At the 3-month follow-up, we did not evaluate for hypercoagulable disorders, since thrombophilia disorders are less likely given her age (> 40 years), a negative personal and family history of VTE, and inadequacy of thrombophilia screening in identifying inherited risks of VTE.

## Discussion and conclusion

Coagulation is a fundamental part of hemostasis. The coagulation cascade represent an equilibrium between the procoagulant pathway responsible for thrombus formation and the anticoagulant pathway that prevent the excessive formation of thrombus. Due to diverse risk factors particularly in hospitalized or critically ill patients, an imbalance in either pathway can result in excessive thrombosis or bleeding. One study investigated the protease activity of *M. oleifera* leaf extract on the coagulation cascade and found that *M. oleifera* selectively hydrolyzes the Aα and Bβ chains of fibrinogen subunits at the N-terminal disulfide leading to clot formation and decreased clotting time from 180 ± 10 s to 119 ± 8 s [9]. Likewise, another study demonstrated a dose-dependent acceleration of clotting time in test subjects who took *M. oleifera* leaves extract when compared to controls (1.64 times shorter in 100% concentrate, 1.27 times shorter in 50% concentrate, and 1.08 times shorter in 25% concentrate) [10]. The observed clot formation activity of *Moringa* in both studies, might be the basis for its reported wound healing properties [9].

Our case report described a rare incidence of sub-massive PE likely provoked by *M. oleifera* leaf extract that have been reported by two studies to have procoagulant activities in human plasma in a patient who had no clear predisposing factor for acute pulmonary embolism. We believe this case report illustrates the importance of considering *M. oleifera* leaf extract as a predisposing factor of PE, that is known chiefly for its health benefits. While, clot formation does not always translate to clot burden; the high fatality rate associated with PE [11, 12], makes future cohort studies necessary to determine the relationship between *Moringa Oleifera* leaf extract and DVT/PE. Adequate data from longitudinal studies will assist clinicians in providing appropriate guidance to patients contemplating the use of the *Moringa Oleifera* leaf extract as a herbal supplement.

## Data Availability

Data sharing is not applicable to this article as no datasets were generated or analyzed for this case report.

## Abbreviations

AC: Anticoagulation
CTPA: Computerized tomography pulmonary angiogram
CXR: Chest X-ray
DVT: Deep vein thrombosis
EKG: Electrocardiogram
ER: Emergency room
ICU: Intensive care unit
PE: Pulmonary embolism
PoCUS: Point of care ultrasound
RV: Right ventricle
US: Ultrasound
VTE: Venous thromboembolism
